# A Novel Model Incorporating Tumor Stiffness, Blood Flow Characteristics, and Ki-67 Expression to Predict Responses After Neoadjuvant Chemotherapy in Breast Cancer

**DOI:** 10.3389/fonc.2020.603574

**Published:** 2020-12-08

**Authors:** Jing Zhang, Song Gao, Qiaojin Zheng, Ye Kang, Jianyi Li, Shuo Zhang, Cong Shang, Xueying Tan, Weidong Ren, Yan Ma

**Affiliations:** ^1^ Department of Ultrasound, Shengjing Hospital of China Medical University, Shenyang, China; ^2^ Department of Clinical Oncology, Shengjing Hospital of China Medical University, Shenyang, China; ^3^ Department of Pathology, Shengjing Hospital of China Medical University, Shenyang, China; ^4^ Department of Breast Surgery, Shengjing Hospital of China Medical University, Shenyang, China; ^5^ Department of Neurology, Shengjing Hospital of China Medical University, Shenyang, China

**Keywords:** blood flow, breast cancer, Neoadjuvant chemotherapy, predictive value, shear-wave elastography

## Abstract

**Objective:**

To investigate the ability of tumor stiffness, tumor blood flow, and Ki-67 expression alone or in combination in predicting the pathological response to neoadjuvant chemotherapy (NACT) in breast cancer.

**Patients and Methods:**

This prospective cohort study included 145 breast cancer patients treated with NACT. Tumor stiffness (maximum stiffness (Emax), mean stiffness (Emean)), blood score (BS), and their relative changes, were evaluated before (t0), during (t1–t5), and at the end of NACT (t6) by shear-wave elastography and optical imaging. Ki-67 expression was quantitatively evaluated by immunohistochemistry using core biopsy specimens obtained before NACT. Pathological responses were evaluated by residual cancer burden. The ability of tumor stiffness, BS, Ki-67, and predRCB—which combined ΔEmean (t2) (the relative changes in Emean after the second NACT cycle), BS2 (BS after the second NACT cycle), and Ki-67—in predicting tumor responses was compared using receiver operating characteristic curves and the Z-test.

**Results:**

Tumor stiffness and BS decreased during NACT. ΔEmean (t2), BS2, and Ki-67 had better predictive performance than other indexes in identifying a favorable response (AUC = 0.82, 0.81, and 0.80) and resistance responses (AUC = 0.85, 0.79, and 0.84), with no significant differences between the three (*p* > 0.05). PredRCB had better predictive performance than any parameter alone for a favorable response (AUC = 0.90) and resistance (AUC = 0.93).

**Conclusion:**

Tumor stiffness, BS, and Ki-67 expression showed good and similar abilities for predicting the pathological response to NACT, and predRCB was a significantly better predictor than each index alone. These results may help design therapeutic strategies for breast cancer patients undergoing NACT.

## Introduction

Neoadjuvant chemotherapy (NACT) is widely used for the treatment of large or locally advanced breast cancer (BC). The response to NACT differs among individuals. Pathological complete response (pCR) is a significant predictor of overall survival and disease-free survival in BC, and approximately 20% of patients achieve pCR after NACT (1). However, certain risk factors ma*y* lead to chemotherapy resistance (2). The ability to predict non-responders in the early stages of chemotherapy would allow response-guided modification of treatment, thereby improving survival outcomes. However, identifying robust predictors of the response to NACT remains challenging because of the use of multiple drug combinations, the genetic variability of tumors, and the variability of outcome measures in available studies.

The tumor response to NACT is determined by intricate interactions between tumor cells and the surrounding microenvironment. Ki-67 is a nuclear antigen that is widely used as a marker of cellular proliferation (3). It is expressed in all phases of the cell cycle except in quiescent cells in the G0 phase. Increased Ki-67 expression, which indicates high proliferative activity, before NACT is a clinical predictor of NACT responses in BC (3–5). However, the predictive value of a single indicator remains controversial (3, 4).

The composition of the extracellular matrix (ECM) also plays an important role in the response to NACT. The ECM is composed of proteins, proteoglycans, and glycosaminoglycans, which mainly include collagen (types I, III, and IV), laminin, elastin, fibronectin, and hyaluronan (HA) (6). Two particularly important molecules, collagen and HA, contribute to matrix stiffness, which is especially important for targeting mechanotransduction pathways in cancer (6, 7). Specifically, in tumor microenvironment with high matrix stiffness, an abnormal collagen composition or increased HA content can increase colloid osmotic pressure, which may lead to increased interstitial fluid pressure (IFP). High IFP can cause distortion and collapse of tumor blood vessels, limiting the uptake of therapeutic drugs that are transported through the vasculature (8, 9), thereby leading to poor response to chemotherapy. High tumor stiffness, as measured by shear-wave elastography (SWE), is closely correlated with chemoresistance in BC (10–12), although more research is needed.

Angiogenesis (or neovascularization), as another important factor in the tumor microenvironment, is considered a rate-limiting step in BC progression, and carries prognostic significance (13). Several imaging modalities yielding different imaging-derived hemodynamic parameters have been proposed for the assessment of tumor vascularity and the response to treatment (14–21). However, dynamic contrast-enhanced magnetic resonance imaging (MRI), and positron emission tomography with computed tomography (PET–CT) have practical constraints, including cost, the use of contrast agents, and exposure to ionizing radiation from PET; in addition, these techniques are not feasible or are contraindicated in some patients, which limits the frequency of monitoring during treatment (16, 17, 19). Optical imaging is a noninvasive technique based on the use of near-infrared (NIR) light to detect blood-volume variations in the microvascular bed of biological tissues. Blood perfusion variations evaluated by optical imaging show good predictive power regarding pCR to NACT in BC patients (17, 19, 21). The effect of chemotherapeutic drug may account for this finding, because the primary effect of most chemotherapeutic drugs is to shrink the tumor, disrupt microvessels, and decrease microvascular density (16, 17). Therefore, for chemosensitive tumors, more total cells are killed and more tumor neovasculature damage occurs, which may cause a significant decrease in tumor hemoglobin measured by an optical imaging system (21).

Tumor stiffness, blood flow characteristics, and Ki-67 expression are early predictors of the pathological response to NACT (3, 10–12, 17, 19). However, to the best of our knowledge, these parameters have not been directly compared to date. Here, we analyzed 145 patients who were treated with NACT to determine the ability of tumor stiffness, blood flow parameters, and Ki-67 expression alone or in combination to predict the response to NACT in BC.

## Materials and Methods

### Patients

Between March 2014 and May 2020, 158 consecutive consenting female patients with newly diagnosed invasive BC who were candidates for NACT and subsequent surgical intervention were prospectively enrolled. The inclusion criteria were as follows: women aged between 18 and 70 years; newly diagnosed to have locally advanced BC (stage II and III); NACT as initial treatment without radiation therapy or endocrine therapy; with complete NACT treatment and all SWE and optical imaging evaluations before surgical intervention; and with pathological diagnosis and NACT response assessment after surgery. Exclusion criteria were as follows: inflammatory BC, metastasis, previous BC, other malignant tumor history, or with severe comorbidity; any contraindication to chemotherapy (poor clinical status, pregnant females) or prior administration of chemotherapy; and unavailability of image data [lesion maximum diameter ≥10 cm and/or deeper than 4 cm on ultrasound (US)].

Among the 158 patients enrolled, 13 patients were excluded: 5 due to change in treatment plans (2 patients proceeded directly to surgery after two NACT cycles due to disease progression and 3 patients underwent presurgical neoadjuvant endocrine therapy) and 8 due to unavailability of image data (5 patients’ lesions with maximum diameter ≥10 cm and 3 patients’ lesions with maximum lesion depth ≥ 4 cm). Thus, 145 patients (mean age, 48.5 years; age range, 30–70 years) were included in the analysis. The study was conducted with the approval of the ethics committee of Shengjing Hospital of China Medical University. All study subjects provided written informed consent.

### Chemotherapy Regimen

Before the surgical procedures, all enrolled eligible patients (*n* = 145) had received six cycles of chemotherapy: 100 received an epirubicin+docetaxel-based regimen; 7 received an epirubicin-based regimen; 6 received a docetaxel-based regimen; 3 received a docetaxel + cisplatin-based regimen, and 29 classified as HER2þ also received Herceptin (trastuzumab) starting on the first cycle of the docetaxel-based therapy.

### SWE and Dynamic Optical Breast Imaging Evaluation

A double-blind SWE and dynamic optical breast imaging (DOBI) evaluation was performed independently by two board-certified radiologists who were blinded to the clinicopathological factors and treatment details during the entire process. SWE and DOBI examination and data analyses started with a training phase, in which images from at least 20 cases were examined. The training phase was supervised by two professional technicians according to the manufacturers’ standard operating procedures.

### Tumor SWE Stiffness Evaluation

The supersonic *Aixplorer* ultrasound system (Supersonic Imagine, Aix en Provence, France) equipped with a 15-4 MHz linear transducer was used to evaluate tumor stiffness. The maximum tumor diameter was measured on the gray-scale US images. Subsequently, four SWE images, two in each of two orthogonal planes of adequate quality, were obtained for each lesion. The criteria for adequate quality were that images displayed abnormal stiffness within the plane without movement or pressure artifacts. A semi-transparent color map of tissue stiffness (color blue, soft; color red, hard) was overlaid on the gray-scale image. The region of interest (ROI) trace (Q-box trace) for the images was selected, including the lesion and the surrounding normal tissue and excluding the chest wall and the skin. Finally, the imaging system automatically calculated the maximum elasticity (Emax) and mean elasticity (Emean) values in kilopascals (range: 0–300 kpa). The average results from the four images were calculated and used for subsequent analyses.

### Tumor Blood Flow Characteristics Evaluation

The blood scores (BS) of the tumors were evaluated using the DOBI system TM-A02 (TRKM Medical Technology Co., LTD, Shenzhen, China). This system is equipped with a near-infrared camera (sensitivity: 0.001–0.01 Lux; resolution: 570–600 lines) and a high-intensity probe (with a dual-wavelength LED illuminator at 730 and 850 nm). BS evaluation was performed in a dark room. Subjects were seated at a distance of 55–75 cm in front of the monitor with the upper body exposed. The DOBI probe was placed on the breast to be examined (in the skew-symmetric quadrant^1^ of the target quadrant to obtain clear images). During the procedure, the probe was attached to the breast skin to prevent light leakage and to ensure that it remained in place for 3–5 s to complete each image acquisition. Subsequently, a two-dimensional distribution image and a functional image were generated by the system. The ROI of the system was set to include the entire lesion on the functional image, which was automatically visualized using a color-coded map with a BS range from green to red (0–4). Then, the BS of the lesion was automatically calculated by the system. BS represents the relative blood volume of the ROI. The standard reference value of BS is 1 (set by the DOBI system); High BS: BS > 1.5; Moderate BS: 0.9 < BS < 1.2; and Low BS < 0.9. For each lesion, the average results obtained from the four images were used for subsequent analysis.

Assessment of tumor stiffness and BS was performed 1 day before chemotherapy (time-point t0, tumor stiffness E0; BS0), 1 day before the next cycle of chemotherapy (t1–t5, E1–E5; BS1–BS5), and after completion of chemotherapy, approximately 1–2 days before surgery (t6, E6; BS6). The relative percentage changes in tumor stiffness and BS were calculated as follows:

$$\begin{array}{l} \Delta Emax\;(ti)=100\%\times [Emax\;(ti)-Emax(t0)]/Emax(t0),\\ \Delta Emean\;(ti)=100\%\times [Emean\;(ti)-Emean(t0)]/Emean(t0),\\ \;\Delta BS\;(ti)=100\%\times [BS\text{ (}ti)-BS(t0)]/BS(t0), \end{array}$$

where i is 1, 2, or 6.

### Pathology and Immunohistochemistry

The pathological evaluation process was divided into two main steps as follows:

First, patients underwent ultrasound-guided tumor biopsy for histological assessment. For immunohistochemical staining, we used the following antibodies: anti-ER (Clone SP1), anti-PR (Clone 1E2), and anti-HER2 (Clone 4B5) from Roche; anti-Ki-67(dilution 1:200; Clone SP6) from Abcam. The immunohistochemistry staining procedure was performed according to the manufacturer’s instructions. For ER and PR, nuclear staining in ≥1% of the tumor cells was considered positive (22). HER2-positivity was defined as tumor cell membrane staining intensity of 3+ or 2+ and/or fluorescence *in situ* hybridization (FISH)-positivity (23). Cells with positive Ki-67 expression were counted based on the percentage of cells with positive nuclear staining among at least 1000 tumor cells. If the staining was homogenous throughout the section, three random high-power fields were selected and the score was calculated. If hot spots were present, then the entire section was analyzed and the overall average score was obtained (3). Lesions were divided into four subtypes as follows: luminal A; luminal B; triple-negative; and HER2 according to the St. Gallen International Expert Panel consensus (24). The histological classification and tumor grade and stage were determined according to internationally recognized guidelines (25–27).

Second, after surgery, the specimens (breast and axillary lymph nodes) were used to evaluate NACT responses using the MD Anderson residual cancer burden (RCB) method (http://www3.mdanderson.org/app/medcalc/index.cfm?pagename=jsconvert3) (28). The RCB score was further classified as RCB-0 (pCR, RCB = 0), RCB-I (0.5 < RCB ≤ 1.36), RCB-II (1.36 < RCB ≤ 3.28), and RCB-III (RCB > 3.28) (28). RCB-0 or RCB-I represented a favorable response (pathological good responders), and RCB-III represented resistance to NACT (non-responders).

### Statistical Analysis

Statistical analysis was performed using SPSS 23.0 software (IBM, USA), Sigmaplot 14.0 (Systat Software, USA), GraphPad Prism 5.0 (GraphPad Software, USA), and MedCalc 15.8 (MedCalc Software, Ostend, Belgium). Continuous variables with skewed distribution were presented as the median and interquartile range, normal distribution was presented as mean ± standard deviation, and categorical variables were presented as *n* (%). Analysis of variance (ANOVA), Kruskal-Wallis test, and χ^2^ test, with post-hoc analysis using LSD, Nemenyi, and Bonferroni tests, respectively, were used to compare the differences among different response groups. The intra-class correlation coefficient (ICC) was computed to assess the inter-observer reproducibility of E0mean, E0max, and BS0. Repeated measures ANOVA was performed to analyze overall differences in tumor stiffness and BS among different groups at different time points. To determine the relationships between SWE stiffness, BS, Ki-67, and RCB scores, general linear models were designed. The area under the ROC curve (AUC) values were drawn to evaluate the predictive power of SWE stiffness, BS, and Ki-67. Finally, a new predictive indicator (predRCB) was developed by combining the predictors with the largest AUC (SWE stiffness and BS) and the traditional marker (Ki-67) according to the results of the multivariable linear regression model. Comparisons between the largest AUC values for different models were performed using the Z-test. Subgroup analysis was conducted to investigate the predictive power of predRCB for both favorable and resistant outcomes according to different characteristics (Molecular subtype, Pathological type, Clinical stage, Grade, and NACT regimen), and the between-subgroup differences were also tested. A two-tailed *p* value of <0.05 was considered significant.

## Results

### Patient and Baseline Characteristics

**Figure 1:**
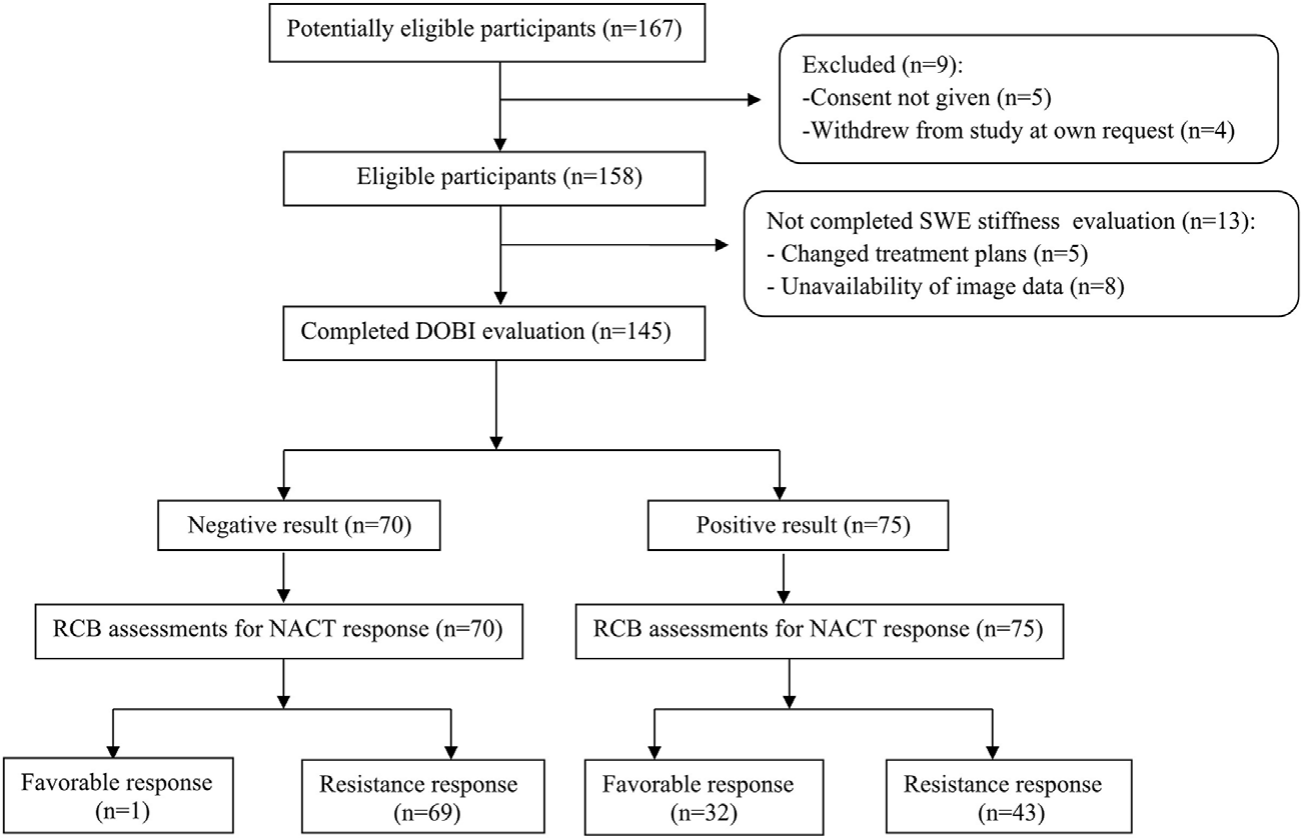
STARD flow chart of patient inclusion. BS2 in ‘DOBI evaluation’ for the prediction of favorable responses is used as an example.

The STARD flow chart in **Figure 1** depicts the selection process for the included studies. Clinical characteristics of the enrolled patients are summarized in **Supplementary Table S1**. Among the 145 patients analyzed, 33 (22.7%) had a favorable response (pCR or RCB-I), 59 (40.7%) had a moderate response (RCB-II), and 53 (36.6%) had NACT resistance (RCB-III). Ki-67, ER positivity, PR positivity in ‘Immunohistochemical marker’, molecular subtype, and clinical stage were different among the three groups (*p* < 0.05). The results indicated that these indicators may have predictive value. However, only Ki-67 can be used for ROC analysis because it is a continuous variable. **Table 1** shows the baseline characteristics.

**Table 1:** Baseline characteristics in different response groups.

Characteristic	Total	Favorable	Moderate	Resistance	F/X2	*p* value
RCB scores	2.21 (1.46–3.70)	0.0 (0.0–1.19)	2.07 (1.78–2.70)	3.94 ± 0.40	125.861	<0.001^#a,b,c^
Patients number	145	33	59	53		
Age (years)	48.50 ± 10.03	46.0 (43.0–53.5)	46.73 ± 10.34	50.94 ± 10.49	4.810	0.09^#^
Maximum diameter (cm)	3.50 (2.55–4.70)	4.00 (2.40–5.05)	3.20 (2.50–4.50)	3.50 (2.65–4.95)	0.764	0.682^#^
Immunohistochemical marker						
Ki-67 (%)	34.90 ± 20.54	53.52 ± 20.90	37.61 ± 16.93	20.28 ± 11.58	43.823	<0.001^¥a,b,c^
ER positive, n (%)	85 (58.6)	15 (45.5)	25 (42.4)	45 (84.9)	23.875	<0.001^*^b,c^^
PR positive, n (%)	79 (54.5)	13 (39.4)	23 (39.0)	43 (81.1)	23.923	<0.001^*^b,c^^
HER2 positive, n (%)	49 (33.8)	11 (33.3)	24 (40.7)	14 (26.4)	2.543	0.280^*^
Molecular subtype, n (%)					25.608	<0.001^*a,b,c^
Luminal A	17 (11.7)	4 (12.1)	3 (5.1)	10 (18.9)		
Luminal B	68 (46.9)	12 (36.4)	21 (35.6)	35 (66.0)		
Triple negative	33 (22.8)	8 (24.2)	20 (33.9)	5 (9.4)		
HER2 positive	27 (18.6)	9 (27.3)	15 (25.4)	3 (5.7)		
Pathological types n (%)					6.129	0.03^*^a,c^^
Invasive ductal carcinoma	134 (92.4)	28 (84.8)	58 (98.3)	48 (90.6)		
Invasive lobular carcinoma	11 (7.6)	5 (15.2)	1 (1.7)	5 (9.4)		
Clinical stage					33.002	<0.001^#b,c^
IIA	24 (16.6)	11 (33.3)	12 (20.3)	1 (1.9)		
IIB	52 (35.9)	10 (30.3)	30 (50.8)	12 (22.6)		
IIIA	53 (36.6)	9 (27.3)	17 (28.8)	27 (50.9)		
IIIB	5 (3.4)	2 (6.1)	0 (0.0)	3 (5.7)		
IIIC	11 (7.6)	1 (3)	0 (0.0)	10 (18.9)		
Grade					1.82	0.403^#^
Grade 1	1 (0.7)	0 (0.0)	0 (0.0)	1 (1.9)		
Grade 2	124 (85.5)	28 (84.8)	54 (91.5)	42 (79.2)		
Grade 3	20 (13.8)	5 (15.2)	5 (8.5)	10 (18.9)		
NACT regimens:					13.787	0.043^*^b,c^^
Epirubicin+docetaxel-based	100 (69.0)	26 (78.8)	40 (67.8)	36 (64.2)		
Herceptin+docetaxel-based	29 (20.0)	6 (18.2)	16 (27.1)	7 (13.2)		
Docetaxel+cisplatin-based	3 (2.1)	1 (3.0)	0 (0.0)	2 (3.8)		
Epirubicin-based	7 (4.8)	0 (0.0)	1 (1.7)	6 (11.3)		
Docetaxel-based	6 (4.1)	0 (0.0)	2 (3.4)	4 (7.5)		

p < 0.05: ^a^favorable response vs. moderate response; ^b^favorable response vs. resistance response; ^c^moderate response vs. resistance response.

^*^χ2 test.

^#^Kruskal-Wallis test.

^￥^ANOVA test.

### Tumor SWE Stiffness and Blood Scores

**Figure 2:**
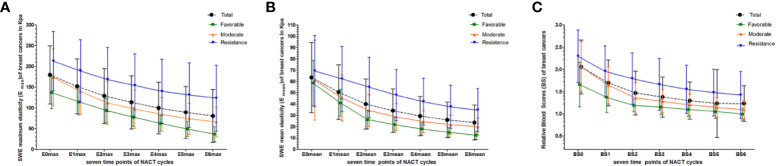
Dynamic changes in Emax, Emean, and BS were measured at seven time points (baseline: t0; interim: t1–t5; and before surgery: t6) for total and different pathological response groups. **(A)** Emax at t0–t6; **(B)** Emean at t0–t6; **(C)** BS at t0–t6.

**Figure 3:**
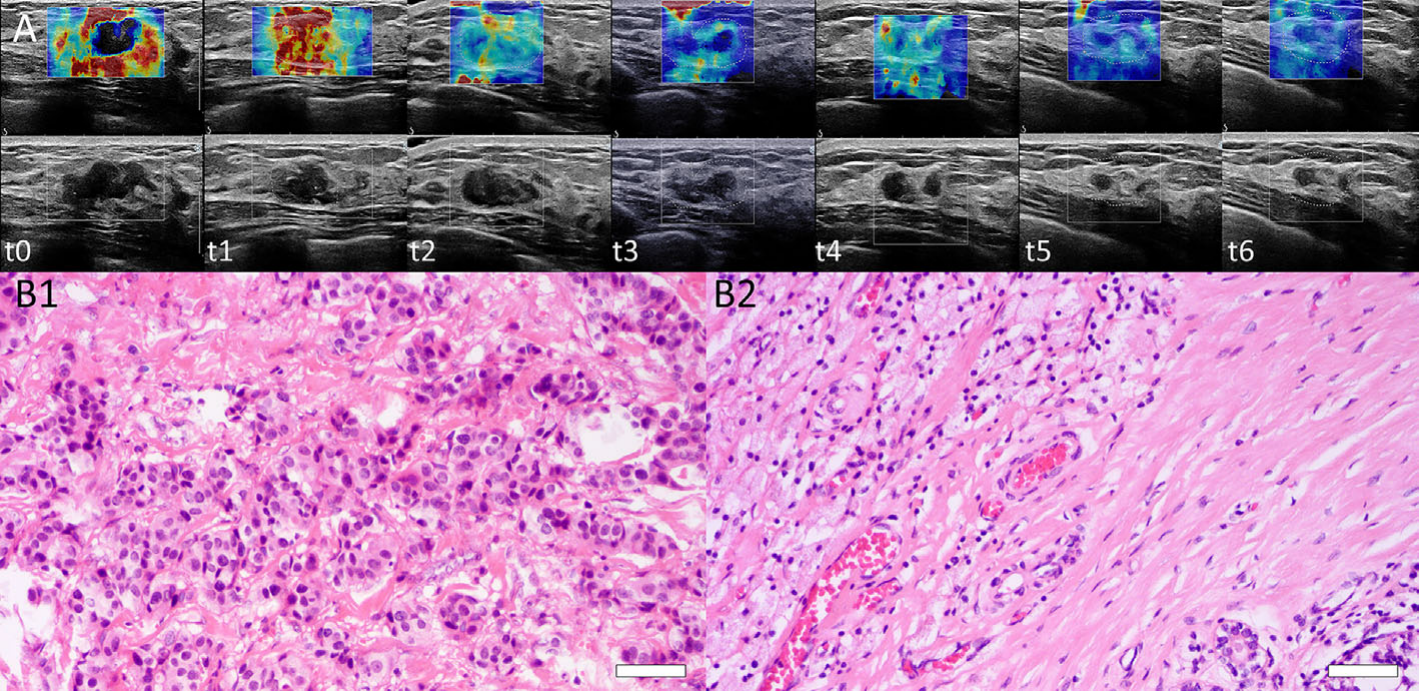
**(A)** Dynamic SWE changes of a lesion at baseline (t0), interim (t1–t5), and before surgery (t6). Emean and Emax were 70.4 and 168.0 kPa, respectively (t0). Tumor stiffness decreased after each NACT cycle. Emean and Emax were 45.4 and 144.4 kPa (t1), 28.0 and 115.0 kPa (t2), 19.4 and 75.2 kPa (t3), 16.0 and 60.3 kPa (t4), and 12.0 and 50.0 kPa (t5), respectively, and 9.8 and 32 kPa before surgery (t6). **(B)** Histopathological identification of patient responses by H&E staining. B1: Before NACT, H&E staining of core needle biopsy tissue from one patient was performed, and invasive ductal carcinoma of the breast was confirmed (H&E stain ×200, Bar = 50μm). B2: After surgery, the surgical specimen of this patient showed a complete histological response with no residual tumor cells remaining (H&E stain ×200, Bar = 50μm). This was a pCR with RCB = 0.

**Figure 4:**
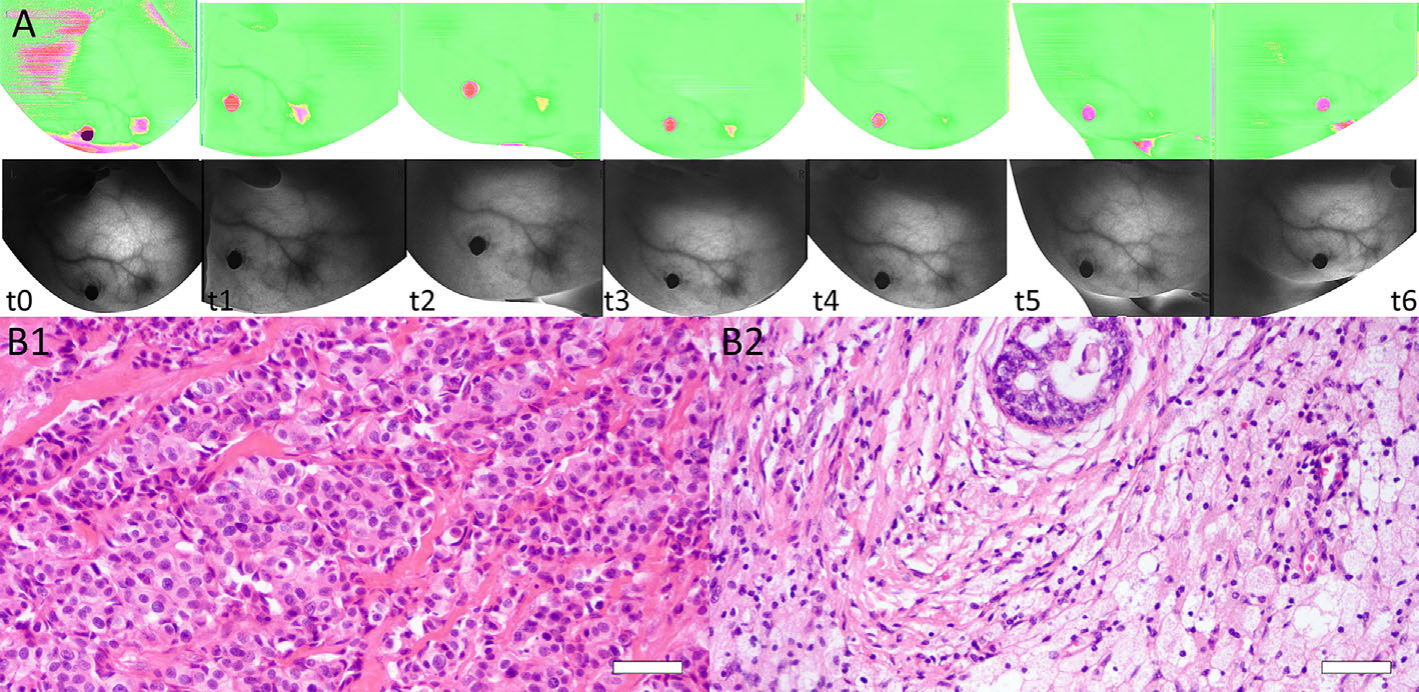
**(A)** Dynamic BS changes of a lesion at baseline (t0), interim (t1–t5), and before surgery (t6). BS was 1.692 at t0 and decreased after each NACT cycle, with values of 1.510 (t1), 1.322 (t2), 1.307 (t3), 1.289 (t4), and 1.112 (t5). BS was 1.074 before surgery (t6). **(B)** Images of tissue specimens of the same breast lesion before and after NACT. B1: Before NACT, the tumor had high cellularity (H&E stain ×200, Bar = 50μm). B2: After surgery, the pathologic result was confirmed as a complete response (RCB = 0) (H&E stain ×200, Bar = 50μm).

The interobserver reliability of E0mean (ICC = 0.87, *p* < 0.001), E0max (ICC = 0.86, *p* < 0.001), and BS0 (ICC = 0.89, *p* < 0.001) was good. SWE stiffness, BS at seven-time points (t0–t6) and the relative changes (ΔEmax, ΔEmean, and ΔBS) in the three groups are presented in **Tables 2** and **3**. SWE stiffness and BS differed significantly between the three groups (*p* < 0.01), except for E0mean and ΔBS (t1) (*p* > 0.05). The repeated measures ANOVA indicated that Emax, Emean, and BS generally differed between the groups at different time points (*F* = 25.72, *p* < 0.001; *F* = 21.32, *p* < 0.001; *F* = 19.81, *p* < 0.001, respectively). Emax, Emean, and BS showed a significant decreasing trend at t0–t6 (**Figure 2**). Representative dynamic changes of SWE images and DOBI images from one lesion during NACT are presented in **Figures 3** and **4**.

**Table 2:** Tumor SWE stiffness (t0-t6) and relative changes (t1, t2, and t6) in different response groups.

SWE stiffness	Total	Favorable	Moderate	Resistance	F	*p* value
E0max	165.8 (135.2–245.1)	138.0 (121.7–146.5)	167.0 (132.0–250.2)	195.0 (143.4–292.9)	26.705	0.001^#a,b,c^
E1max	140.4 (105.6–188.6)	114.48 ± 31.02	139.50 ± 54.41	169.4 (123.0–256.1)	24.761	<0.001^#a,b,c^
E2max	120.5 (80.8–151.6)	97.2 (78.9–117.5)	112.17 ± 51.97	141.7 (114.7–245.1)	26.621	<0.001^#b,c^
E3max	99.3 (70.0–131.6)	77.66 ± 22.67	96.89 ± 46.16	125.4 (98.7–230.3)	31.949	<0.001^#a,b,c^
E4max	85.4 (58.0–115.6)	60.5 (51.5–71.2)	83.99 ± 40.71	113.6 (79.8–215.4)	35.193	<0.001^#a,b,c^
E5max	77.6 (44.4–106.0)	49.13 ± 3.11	79.2 (35.0–100.7)	102.9 (73.3–198.1)	41.439	<0.001^#a,b,c^
E6max	67.2 (31.3–98.9)	30.9 (21.9–46.0)	70.3 (30.1–89.1)	96.5 (61.6–179.1)	46.774	<0.001^#a,b,c^
E0mean	56.2 (41.9–71.3)	52.7 (44.5–67.8)	55.0 (40.1–65.3)	59.9 (46.3–81.9)	4.027	0.134^#^
E1mean	45.0 (35.4–60.5)	41.06 ± 11.86	45.33 ± 20.38	59.0 (41.3–72.0)	17.189	<0.001^#b,c^
E2mean	35.3 (25.9–51.5)	26.25 ± 8.05	34.21 ± 14.18	52.0 (34.8–63.3)	41.919	<0.001^#a,b,c^
E3mean	30.1 (20.7–42.8)	21.89 ± 6.87	28.60 ± 11.75	45.1 (29.7–54.6)	45.584	<0.001^#a,b,c^
E4mean	24.6 (17.9–36.4)	17.99 ± 5.43	24.34 ± 9.79	39.8 (24.6–50.3)	50.474	<0.001^#a,b,c^
E5mean	22.1 (15.3–31.0)	14.98 ± 4.23	21.78 ± 8.44	34.5 (23.3–49.9)	53.889	<0.001^#a,b,c^
E6mean	19.9 (14.4–28.3)	10.9 (10.0–15.1)	19.99 ± 7.76	28.4 (18.7–48.6)	54.423	<0.001^#a,b,c^
ΔEmax (t1)	−0.13 (−0.25 to −0.07)	−0.15 ± 0.09	−0.17 (−0.30 to −0.08)	−0.13 ± 0.12	6.920	0.031^#c^
ΔEmax (t2)	−0.29 ± 0.19	−0.30 (−0.35 to −0.15)	−0.35 ± 0.20	−0.23 ± 0.18	10.316	0.006^#c^
ΔEmax (t6)	−0.60 (−0.79 to −0.44)	−0.79 (−0.83 to −0.65)	−0.65 (−0.78 to −0.47)	−0.43 ± 0.28	29.031	<0.001^#a,b,c^
ΔEmean (t1)	−0.16 (−0.24 to −0.09)	−0.28 (−0.36 to −0.18)	−0.16 (−0.26 to −0.12)	−0.07 (−0.14 to −0.04)	49.258	<0.001^#a,b,c^
ΔEmean (t2)	−0.32 (−0.52 to −0.21)	−0.52 ± 0.16	−0.36 (−0.52 to −0.25)	−0.21 ± 0.12	58.375	<0.001^#a,b,c^
ΔEmean (t6)	−0.63 (−0.75 to −0.50)	−0.76 ± 0.11	−0.63 (−0.72 to −0.55)	−0.49 ± 0.17	47.659	<0.001^#a,b,c^

p < 0.05: ^a^favorable response vs. moderate response; ^b^favorable response vs. resistance response; ^c^moderate response vs. resistance response.

^#^Kruskal-Wallis test.

**Table 3:** BS (t0-t6) and relative changes (t1, t2, and t6) in different response groups.

Blood scores	Total	Favorable	Moderate	Resistance	F	*p* value
BS0	2.12 (1.53–2.46)	1.53 (1.30–1.94)	2.19 (1.53–2.56)	2.30 (1.96–2.74)	23.173	<0.001^#a,b^
BS1	1.67 (1.31–1.91)	1.29 (1.13–1.57)	1.64 ± 0.39	1.80 (1.61–2.22)	24.560	<0.001^#a,b,c^
BS2	1.38 (1.17–1.69)	1.11 (1.05–1.30)	1.38 ± 0.33	1.63 (1.43–1.93)	44.703	<0.001^#a,b,c^
BS3	1.30 (1.13–1.52)	1.12 ± 0.15	1.30 ± 0.25	1.42 (1.30–1.71)	45.800	<0.001^#a,b,c^
BS4	1.23 (1.06–1.42)	1.05 ± 0.16	1.22 (1.05–1.33)	1.35 (1.25–1.59)	46.087	<0.001^#a,b,c^
BS5	1.16 (1.01–1.36)	1.01 (0.86–1.09)	1.15 ± 0.21	1.30 (1.18–1.49)	47.850	<0.001^#a,b,c^
BS6	1.10 (1.00–1.30)	0.96 (0.82–1.04)	1.09 (1.01–1.23)	1.29 (1.10–1.45)	45.974	<0.001^#a,b,c^
ΔBS (t1)	−0.16 (−0.25 to −0.07)	−0.15 ± 0.09	−0.19 ± 0.10	−0.16 (−0.26 to −0.04)	4.99	0.083^#^
ΔBS (t2)	−0.27 ± 0.01	−0.25 ± 0.13	−0.31 ± 0.15	−0.23 ± 0.13	5.08	0.007^¥c^
ΔBS (t6)	−0.43 (−0.51 to −0.32)	−0.38 (−0.46 to−0.31)	−0.45 ± 0.12	−0.37 ± 0.15	9.092	0.011^#a,b^

p < 0.05: ^a^favorable response vs. moderate response; ^b^favorable response vs. resistance response; ^c^moderate response vs. resistance response.

^#^Kruskal-Wallis test.

^￥^ANOVA test.

### Relationships Between Tumor Stiffness, BS, Ki-67, and RCB Scores

SWE stiffness, BS, and Ki-67 showed significant independent relationships with RCB scores (*R^2^* range 0.163–0.391, *p* < 0.01), except for ΔEmax (t1, t2), E0mean, and ΔBS (t1, t2, t6). Except for values at t6, ΔEmean (t2) in SWE stiffness, BS2 in BS parameters, and Ki-67 maintained the highest proportion of variation in RCB scores (*R* ^2^ = 0.332, 0.282, and 0.326, respectively), which indicated that these parameters had a significant independent relationship with pathological responses. **Table 4** presents the results of the general linear model.

**Table 4:** The relationship among tumor stiffness, BS, Ki-67, and RCB scores assessed by general linear models.

Parameters	R^2^	F-ratio	*p* value
E0max	0.186	32.705	<0.001
E1max	0.226	41.808	<0.001
E2max	0.231	42.964	<0.001
E6max	0.372	84.659	<0.001
E0mean	0.043	6.400	0.012
E1mean	0.163	27.892	0.001
E2mean	0.300	61.196	<0.001
E6mean	0.391	91.835	<0.001
ΔEmax (t1)	0.032	4.743	0.031
ΔEmax (t2)	0.045	6.721	0.011
ΔEmax (t6)	0.262	50.664	<0.001
ΔEmean (t1)	0.187	32.946	<0.001
ΔEmean (t2)	0.332	70.922	<0.001
ΔEmean (t6)	0.307	63.267	<0.001
BS0	0.177	30.629	<0.001
BS1	0.205	36.842	<0.001
BS2	0.282	56.074	<0.001
BS6	0.023	3.334	0.07
ΔBS (t1)	0.000	0.004	0.834
ΔBS (t2)	0.021	3.095	0.081
ΔBS (t6)	0.007	1.068	0.303
Ki-67	0.326	69.119	<0.001

### Predictive Performance of SWE Stiffness, BS, and Ki-67

The AUCs and 95% confidence intervals of SWE stiffness, BS, and Ki-67 for predicting NACT responses are summarized in **Table 5**. Among these indexes, ΔEmean (t2), BS2, and Ki-67 showed the best predictive performance for both a favorable response (AUC = 0.82, 0.81, and 0.80, respectively) and resistance (AUC = 0.85, 0.79, and 0.84, respectively). According to the Z-test, no significant differences in AUC were observed between these indexes (*p* > 0.05). These results suggest that tumor SWE stiffness, BS, and Ki-67 have a good and similar predictive power for different NACT responses.

**Table 5:** Performance of tumor stiffness, BS, and Ki-67 expression for predicting different responses to NACT.

	Favorable response			Resistance response		
	AUC (95%Cl)	SE	*p* value	AUC (95%Cl)	SE	*p* value
Tumor Stiffness						
E0max	0.75 (0.67–0.83)	0.041	<0.001	0.72 (0.63–0.80)	0.046	<0.001
E1max	0.73 (0.65–0.81)	0.041	<0.001	0.72 (0.63–0.81)	0.046	<0.001
E2max	0.71 (0.63–0.79)	0.042	<0.001	0.74 (0.66–0.83)	0.044	<0.001
E0mean	0.53 (0.43–0.64)	0.052	0.552	0.60 (0.50–0.70)	0.049	0.046
E1mean	0.66 (0.56–0.75)	0.047	0.006	0.70 (0.61–0.79)	0.045	<0.001
E2mean	0.78 (0.71–0.85)	0.038	<0.001	0.80 (0.72–0.87)	0.040	<0.001
ΔEmax (t1)	0.50 (0.40–0.60)	0.052	0.966	0.62 (0.52–0.72)	0.049	0.016
ΔEmax (t2)	0.51 (0.41–0.61)	0.052	0.873	0.65 (0.56–0.74)	0.047	0.003
ΔEmean (t1)	0.79 (0.71–0.86)	0.039	<0.001	0.83 (0.75–0.90)	0.037	<0.001
ΔEmean (t2)	0.82(0.74–0.89)	0.039	<0.001	0.85 (0.79–0.92)	0.031	<0.001
Tumor blood flow						
BS0	0.76 (0.67–0.85)	0.046	<0.001	0.68 (0.59–0.76)	0.045	0.001
BS1	0.75 (0.65–0.84)	0.049	<0.001	0.70 (0.62–0.79)	0.044	<0.001
BS2	0.81 (0.74–0.89)	0.037	<0.001	0.79 (0.71–0.86)	0.039	<0.001
ΔBS (t1)	0.58 (0.48–0.68)	0.051	0.152	0.55 (0.45–0.65)	0.053	0.327
ΔBS (t2)	0.55 (0.44–0.65)	0.055	0.411	0.62 (0.52–0.71)	0.049	0.081
Traditional marker						
Ki-67	0.81 (0.72–0.89)	0.042	<0.001	0.84 (0.78–0.90)	0.032	<0.001
Combined						
PredRCB	0.90 (0.86–0.95)	0.024	<0.001	0.93 (0.90–0.97)	0.019	<0.001

Next, we tested whether combining the three parameters could improve the performance for predicting NACT responses. Toward this end, ΔEmean (t2), BS2, and Ki-67 were combined into a new predictive index (predRCB), which was based on the multivariable linear regression model (predRCB = 3.147–0.025 × Ki-67 + 2.400 × ΔEmean (t2) + 0.688 × BS2). PredRCB showed a fairly good predictive power for a favorable response (AUC = 0.90) and resistance to NACT (AUC = 0.93).

**Figure 5:**
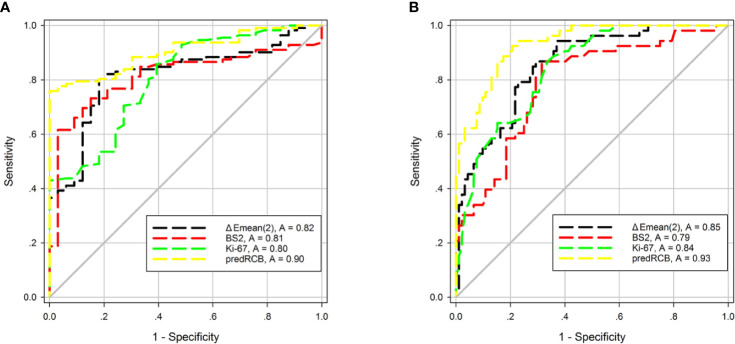
ROC curves of tumor stiffness, BS, Ki-67, and predRCB for predicting **(A)** a favorable response and **(B)** a resistance response.

In the prediction of a favorable response or resistance to NACT, the AUCs differed significantly between predRCB and other indexes alone according to the Z-test (*p* < 0.05). This indicates that predRCB was a better predictor than the single parameter (tumor stiffness, BS, or Ki-67) (**Figure 5**). **Table 6** shows the optimum cut-off values of ΔEmean (t2), BS2, Ki-67, and predRCB for predicting different responses, and predRCB displayed the best predictive ability when the values were set at 2.3020 for a favorable response and 2.5460 for a resistance response.

**Table 6:** Cutoffs and predictive diagnostic performance for different responses to NACT.

		Cut-offs	Sens (%)	95% CI	Spec (%)	95% CI
Favorable response	Ki-67	≥47.50	60.61	42.14–77.09	85.71	77.84–91.61
	ΔEmean (t2)	≤−0.4143	81.82	64.54–93.02	80.36	71.78–87.26
	BS2	≤1.400	96.97	84.24–99.92	61.61	51.94–70.64
	PredRCB	≤2.302	100.00	89.42–100.00	75.89	66.90–83.49
Resistance response	Ki-67	<33.50	86.79	74.66–94.52	66.30	55.70–75.83
	ΔEmean (t2)	>–0.3236	86.79	74.66–94.52	70.65	60.24–79.69
	BS 2	>1.3720	86.79	74.66–94.52	68.48	57.96–77.77
	PredRCB	>2.5460	92.45	81.79–97.91	79.35	69.64–87.08

### Predictive Performance of predRCB According to Subtype

The results of subgroup analyses are shown in **Table 7**. PredRCB showed good predictive ability for both favorable and resistant outcomes (AUC > 0.75) in different subgroups (Molecular subtype, Pathological type, Clinical stage, Grade, and NACT regimen).

**Table 7:** Predictive diagnostic performance of predRCB in the different subgroups.

Subtype	Favorable response				Resistance response			
	AUC(95%Cl)	SE	*p* value	*p*_group_	AUC(95%Cl)	SE	*p* value	*p*_group_
Pathological type				<0.05				<0.05
Invasive ductacarcinoma	0.89 (0.83–0.94)	0.028	<0.001		0.92 (0.88–0.97)	0.022	<0.001	
Invasive lobular carcinoma	1.00 (1.00–1.00)	0.00	0.006		1.00 (1.00–1.00)	0.00	0.006	
Molecular subtype				<0.05				>0.05
Luminal A	1.00 (1.00–1.00)	0.000	0.003		0.96 (0.87–1.00)	0.045	0.001	
Luminal B	0.88 (0.80–0.96)	0.043	<0.001		0.94 (0.88–0.99)	0.029	<0.001	
Triple negative	0.98 (0.95–1.00)	0.019	<0.001		0.92 (0.79–1.00)	0.066	0.001	
HER2	0.77 (0.59–0.95)	0.091	0.024		0.92 (0.76–1.00)	0.078	0.021	
Clinical stage				>0.05				>0.05
IIA	–	–	–		0.96 (0.87–1.00)	0.043	0.129	
IIB	0.90 (0.82–0.98)	0.042	<0.001		0.87 (0.75–0.98)	0.060	<0.001	
IIIA	0.96 (0.90–1.00)	0.028	<0.001		0.93 (0.86–0.99)	0.033	<0.001	
IIIB	1.00 (1.00–1.00)	0.00	0.083		1.00 (1.00–1.00)	0.00	0.083	
IIIC	1.00 (1.00–1.00)	0.00	0.114		1.00 (1.00–1.00)	0.00	0.114	
Grade				<0.05				>0.05
Grade 1	–	–	–		–	–	–	
Grade 2	0.89 (0.83–0.94)	0.029	<0.001		0.92 (0.88–0.97)	0.023	<0.001	
Grade 3	0.99 (0.95–1.00)	0.021	0.001		0.97 (0.90–1.00)	0.035	<0.001	
NACT regimen:				>0.05				>0.05
Epirubicin+docetaxel-based	0.92 (0.87–0.97)	0.026	<0.001		0.93 (0.88–0.98)	0.025	<0.001	
Herceptin+docetaxel-based	0.77 (0.59–0.94)	0.09	0.046		0.96 (0.88–1.00)	0.039	<0.001	
Docetaxel+cisplatin-based	1.00 (1.00–1.00)	0.00	0.221		1.00 (1.00–1.00)	0.00	0.221	
Epirubicin-based	–	–	–		1.00 (1.00–1.00)	0.00	0.134	
Docetaxel-based	–	–	–		1.00 (1.00–1.00)	0.00	0.064	

“ – “: values could not be calculated due to the limited sample after subgrouping.

p _group_: Testing the between-subgroup differences.

According to the results of between-subgroup analysis, in the prediction of a favorable response, the Z-test of AUCs indicated that predRCB exhibited no significant differences in AUCs within the ‘Clinical stage’ and ‘NACT regimen’ subgroups (all *p* > 0.05). It suggested that predRCB had good and similar performances in these subgroups. Moreover, predRCB performed better in patients with invasive lobular carcinoma, luminal A-type and triple negative-type in ‘Molecular subtype’, and Grade 3 (all *p* < 0.05).

In the prediction of NACT resistance, all subgroups showed no significant difference in AUCs of predRCB, except for ‘Pathological type’ (*p* < 0.05), indicating that predRCB had better predictive performance in patients with invasive lobular carcinoma (*p* < 0.05) and good and similar performances in ‘Molecular subtype’, ‘Clinical stage’, ‘Grade’, and ‘NACT regimen’ (all *p* > 0.05).

## Discussion

The main results of the present study can be summarized as follows: (i) ΔEmean (t2), BS2, and Ki-67 expression showed a better predictive power than other indexes regarding the pathological responses to NACT. (ii) PredRCB had greater predictive power than ΔEmean (t2), BS2, and Ki-67 alone. (iii) PredRCB showed good predictive ability in the different subgroups (Molecular subtype, Pathological type, Clinical stage, Grade, and NACT regimen).

Ki-67 is a widely used nuclear antigen-specific biomarker of cellular proliferation and a prognostic factor in BC. It has been reported that higher (>25%) or lower (<12%) Ki-67 expression before NACT showed good prediction for pCR or resistance to NACT in BC (29). Recently, Jain et al. suggested that the best cut-off value of Ki-67 to predict pCR is 35% (3). To date, there is no consensus regarding the standard cut-off value of Ki-67 staining. In this study, the expression of Ki-67 was significantly higher in the favorable response group than in the resistance group. These findings are consistent with those from previous studies (3, 5, 29, 30).

In addition to the tumor cells themselves, the tumor microenvironment is another important determinant of chemotherapeutic responses. Ki-67, a well-established marker of cell proliferation, is expressed during all active phases of the cell cycle. Rather than tumor cell features, tumor stiffness and BS predominantly represent the features of the tumor ECM and angiogenesis, respectively. Pretreatment tumor stiffness is statistically significantly related to NACT responses in BC (10–12). Similarly, tumor blood-volume variations observed in the initial 10 days to 2 weeks of NACT by optical imaging show good predictive power (17, 19, 31). However, there are relatively few reports addressing these issues, and more studies are needed. To the best of our knowledge, there are no reports comparing the value of tumor cell features and multiple tumor microenvironmental characteristics for predicting the responses to NACT in BC.

In the present study, we confirmed that BC tumors with lower BS and lower stiffness were associated with better pathological responses (pCR or RCBI), whereas tumors with higher BS and higher stiffness displayed resistance to NACT. These results support the findings of previous studies (10, 12, 14, 15). Moreover, tumor stiffness and BS decreased during NACT for all patients. The effect of the chemotherapy drug may account for this result, because the primary effect of most chemotherapy drugs is to shrink the tumor, cause tumor fibrosis, and disrupt the blood supply (15, 32), thereby leading to a decrease in tissue stiffness and BS. In addition, the relative change rates in SWE stiffness and BS showed a greater decrease in good responders than in non-responders. This can be attributed to NACT-induced changes in biomechanical properties and microvascular perfusion (32, 33). NACT causes fibrosis and interrupts the blood supply in the responding tumor tissue, which leads to a greater decrease in tumor stiffness and BS in good responders than in non-responders.

Next, we used these indexes to predict the pathological responses to NACT in BC. The results showed that tumor stiffness, BS, and Ki-67 had comparable predictive abilities. Clinically, for non-responders, the optimal timing of surgery is after the first or second NACT cycle (10). Thus, in the present study, we chose values at t0–t2 to evaluate the predictive abilities. The results showed that tumor stiffness and BS at t2 had the best performance, which is consistent with the optimal time point reported in previous studies with MRI and PET (34, 35). This finding may be attributed to the following reason: NACT initially alters the biomechanical properties and microvascular perfusion in the tumor, whereas morphological changes that can be observed on imaging occur later (33, 36). Several studies also showed that tumor size variation is not a sensitive index to distinguish responders from non-responders (37).

This study also confirmed that a combination of tumor cells themselves and tumor microenvironment-associated features can improve the predictive performance compared with that of a single factor for the early identification of NACT responses in BC. ΔEmean (t2), BS2, and Ki-67, which performed best for predicting different responses, were combined into a new predictor termed predRCB using a multivariable linear regression model. PredRCB was a better predictor than the other individual indexes for different NACT responses. Furthermore, predRCB displayed good predictive value within the five different subgroups (Molecular subtype, Pathological type, Clinical stage, Grade, and NACT regimen). This provided strong evidence supporting the validity and generalizability of our findings. To the best of our knowledge, this study is the first to show that a combination of tumor cells and multiple tumor microenvironmental characteristics can improve the predictive power of single indexes for NACT responses in BC.

This study had several limitations. First, the relatively small sample size may limit the statistical power of the current study. Our results need to be further validated in a multicenter trial with larger independent cohort. Second, the internal blood vessels and their perfusion, and tissue stiffness, are not evenly distributed within the tumor tissue (as a three-dimensional structure). BS and tumor stiffness were assessed locally, which does not reflect the entire tumor. The use of three-dimensional parameters from the tumor may further improve the predictive ability. Third, the endogenous expression of Ki-67 in the whole tumor was also unevenly distributed. Ki-67 expression was assessed using pathological tissue samples from core needle biopsy, which does not fully reflect tumor heterogeneity. Performing multi-site biopsies and calculating the average results might reduce this difference. Fourth, although subgroup analyses with many participant characteristics were performed, we cannot rule out the possibility that unmeasured factors might contribute to the obtained results. For example, BRCA status was not measured in the present study. Future studies that include more confounding factors are therefore needed. Finally, because of the short duration of follow-up, the overall survival and disease-free survival were difficult to assess. A longer-term follow-up study should be performed to verify the results of the present study.

## Conclusion

Tumor stiffness, BS, and Ki-67 expression exhibited good and similar performances for the early identification of NACT responses in BC. This study highlighted the potential value of predRCB, which improved the predictive ability of each single indicator. The results of the present study may help tailor individualized treatment regimens for BC patients receiving NACT.

## Data Availability Statement

The original contributions presented in the study are included in the article/**Supplementary Materials**, further inquiries can be directed to the corresponding author.

## Ethics Statement

This study involving human participants was reviewed and approved by the ethics committee of Shengjing Hospital of China Medical University. All patients provided written informed consent. The study was conducted according to the Declaration of Helsinki.

## Author Contributions

JZ, SG, and YM contributed to the conception and design of the study. QZ, JL, and YK contributed to the collection and assembly of data. SZ organized the database. CS and XT performed data analysis and interpretation. JZ wrote the first draft of the manuscript. SG, WR, and YM revised the language and reviewed the manuscript. All authors read and approved the final version. All authors contributed to the article and approved the submitted version.

## Funding

This work was supported by the National Natural Science Foundation of China (81801710, 81571686) and the Ph.D. Research Startup Foundation of Liaoning Province (20180540024), 345 Talent Project, Shengjing Hospital of China Medical University.

## Conflict of Interest

The authors declare that the research was conducted in the absence of any commercial or financial relationships that could be construed as a potential conflict of interest.

## Footnotes

1 We place the DOBI probe in the 3rd (4th) quadrant if the target quadrant is the 1st (2nd) quadrant.

## Supplementary Material

Supplementary Table 1 Clinical characteristics of the 145 enrolled patients. Unless noted otherwise, data are reported as number of patients with percentages in parentheses. SD = standard deviation. RCB = residual cancer burden.

## Abbreviations

AUC, area under the ROC; BC, breast cancer; BS, blood score; Emax, maximum elasticity; Emean, mean elasticity; ECM, extracellular matrix; NACT, neoadjuvant chemotherapy; NIR, near-infrared; IFP, interstitial fluid pressure; pCR, pathological complete response; RCB, residual cancer burden; ROC, receiver operating characteristics; ROI, region of interest; SWE, shear-wave elastography; US, ultrasound.
