# Supplementary material for: A Novel Model Incorporating Tumor Stiffness, Blood Flow Characteristics, and Ki-67 Expression to Predict Responses After Neoadjuvant Chemotherapy in Breast Cancer
Source: Front Oncol. 2020 Dec 8;10:603574. doi: 10.3389/fonc.2020.603574 (PMC7753215; doi:10.3389/fonc.2020.603574)
Supplement: Supplementary Table 1 — Clinical characteristics of the 145 enrolled patients. Unless noted otherwise, data are reported as number of patients with percentages in parentheses. SD = standard deviation. RCB = residual cancer burden. [file Table_1.docx]

**[Supplementary Table S1](https://www.ncbi.nlm.nih.gov/pmc/articles/PMC4857102/#S1)**

Clinical characteristics of the 145 enrolled patients

| characteristics | Value |
| --- | --- |
| Age (y), mean±SD  BMI (range)  Menopausal status  Premenopausal  Postmenopausal  Molecular subtype  Luminal A  Luminal B  Triple negative  HER2 positive  Pathological types  Invasive ductal carcinoma  Invasive lobular carcinoma  Initial clinical stage  IIA  IIB  IIIA  IIIB  IIIC  NACT regimen  Epirubicin+docetaxel-based  Herceptin+docetaxel-based  Docetaxel+cisplatin-based  Epirubicin-based  Docetaxel-based  Type of surgery  Breast-conserving  Total mastectomy  Pathologic response (RCB scores)  RCB-0 (pCR)  RCB-I  RCB-II  RCB-III | 48.50 ± 10.03  22.85 (18.36–30.49)  83 (57.2)  62 (42.8)  17 (11.7)  68 (46.9)  33 (22.8)  27 (18.6)  134 (92.4)  11 (7.6)  24 (16.6)  52 (35.9)  53 (36.6)  5 (3.4)  11 (7.6)  100 (69.0)  29 (20.0)  3 (2.1)  7 (4.8)  6 (4.1)  12 (8.3)  133 (91.7)  19 (13.1)  14 (9.7)  59 (40.7)  53 (36.6) |

Note—Unless noted otherwise, data are reported as number of patients

with percentages in parentheses. SD = standard deviation.

RCB = residual cancer burden.
